# Qualitative and Quantitative Phytochemical Analysis of *Ononis* Hairy Root Cultures

**DOI:** 10.3389/fpls.2020.622585

**Published:** 2021-01-13

**Authors:** Nóra Gampe, Zoltán Szakács, András Darcsi, Imre Boldizsár, Éva Szőke, Inna Kuzovkina, László Kursinszki, Szabolcs Béni

**Affiliations:** ^1^Department of Pharmacognosy, Semmelweis University, Budapest, Hungary; ^2^Spectroscopic Research Department, Gedeon Richter Plc., Budapest, Hungary; ^3^Natural Bioactive Compounds Group, Institutional Excellence Program, Department of Plant Anatomy, Eötvös Loránd University, Budapest, Hungary; ^4^Timiryazev Institute of Plant Physiology, Russian Academy of Sciences, Moscow, Russia

**Keywords:** *Ononis*, hairy root, biotechnology, isoflavonoid, bulatlactone, ononilactone

## Abstract

Hairy root cultures are genetically and biochemically stable, and they regularly possess the same or better biosynthetic capabilities for specialized (secondary) metabolite production compared to the intact plant. *Ononis* species are well-known herbal remedies in ethnopharmacology and rich sources of isoflavonoids. Besides isoflavones, less prevalent isoflavones and pterocarpans with valuable biological effects can be found in *Ononis* species as well. As these plants are only collected but not cultivated, biotechnological methods could play a role in the larger-scale extraction of *Ononis* isoflavonoids. Regarding this information, we aimed to establish *Ononis spinosa* and *Ononis arvensis* hairy root cultures (HRCs) and analyze the isoflavonoid profile of hairy root cultures qualitatively and quantitatively, in order to define their capacity to produce biologically valuable isoflavonoids. During the qualitative description, beside isoflavonoids, two new phenolic lactones, namely, bulatlactone 2″-*O*-β-D-glucoside and ononilactone, were isolated, and their structures were characterized for the first time. Altogether, 29 compounds were identified by the means of UPLC-Orbitrap-MS/MS. Based on UHPLC-UV-DAD measurements, the isoflavonoid spectrum of the *Ononis* HRCs differed markedly from wild-grown samples, as they produce a limited range of the scaffolds. The most abundant compounds in the HRCs were medicarpin glucoside and sativanone glucoside. The overall isoflavonoid production of the cultures was comparable to wild-grown *O. arvensis* and approximately twice as high as in wild-grown *O. spinosa* samples. As the overall content of wild-grown samples include more isoflavonoid derivatives, the HRCs contain structurally less divergent isoflavonoids but in higher quantity.

## Introduction

Bioactive natural products are molecules perfected by evolution, which, based on their physico-chemical properties, are much more likely to become potential drug candidates than synthetic compounds produced by combinatorial chemical methods (Larsson et al., 2005). Their sources might be herbs that are easy to cultivate, e.g., lavender or chamomile, etc., but in many cases, it is only possible to isolate it from wild-growing populations, e.g., taxol. This poses a major threat to ecological diversity, habitat undisturbedness, or even the survival of the species. An additional difficulty could be if the intact plant contains the active compound only as a minor component, and its extraction is not economical (Atanasov et al., 2015).

*In vitro* cultivation of medicinal plants can provide a solution to this problem. Micro-propagation, organ, and cell cultures present an opportunity to produce the desired species under laboratory conditions and to bioreplicate individuals and/or organs with the highest active substance content. Although plant cell cultures seemed to be very promising tools in the production of specialized metabolites, unfortunately, they often did not live up to their expectations. The reasons can lie in the difficulty of industrial upscaling. Another problem is that the lack of differentiation and compartmentalization, that can result in different metabolic profiles compared to the intact plant (Atanasov et al., 2015). Hairy root cultures, as transgenic tissue cultures, show a higher degree of differentiation. These are created by the bacterial infection of *Agrobacterium rhizogenes*, during which bacterial plasmid is incorporated into the plant DNA causing prolific growth of neoplastic roots. Hairy root systems can be maintained without the use of phytohormones. As opposed to cell cultures, they are genetically and biochemically stable, and they regularly possess the same or better biosynthetic capabilities for specialized metabolite production as the intact plant (Georgiev et al., 2012).

Isoflavonoids are products of the phenylpropanoid biosynthesis route, and their main groups are isoflavones, isoflavanones, and pterocarpans (Davies and Schwinn, 2006). Their best-known representatives are compounds belonging to the group of isoflavones, which are found in food and agricultural crops such as soy, alfalfa, or red clover. These compounds are used mainly because of their phytoestrogenic effect, and since the wild-grown plants are cultivated in large areas, they are easy to obtain (Clifford and Brown, 2006). In addition to isoflavones, the group of isoflavanones and pterocarpans also include compounds with valuable biological effects; however, the plants that contain them mostly live only in the wild. For example, *Ononis* species are well-known herbal remedies in the Mediterranean region. As a member of the Fabaceae (*Leguminosae*) family, they are rich in isoflavonoids, and beside isoflavones, they produce less-prevalent isoflavanones and pterocarpans (Gampe et al., 2016, 2018b). Despite the medicinal benefits (Addotey et al., 2018; Deipenbrock et al., 2020; Spiegler et al., 2020; Stojkovic et al., 2020; Stojković et al., 2020), the plant is not cultivated. The part of the herb mostly used in phytomedicine is the extremely hardy root, making the collection cumbersome. Regarding these aspects, biotechnological methods could play a role in the larger-scale yield of *Ononis* isoflavonoids. Tumova et al. investigated the flavonoid content of callus cultures and cell suspensions of *Ononis arvensis*, but their experiments covered only the measurement of total flavonoid content in elicited cultures (Tůmová and Rusková, 1998; Tůmová et al., 2003, 2011; Tůmová and Polívková, 2006). Regarding this information, we aimed to analyze the isoflavonoid profile of hairy root cultures qualitatively and quantitatively, in order to define their capacity to produce biologically valuable isoflavonoids.

## Materials and Methods

### General Materials

Standard compound naringenin were purchased from Sigma-Aldrich (St. Louis, MO, United States), and formononetin, pseudobaptigenin, onogenin, sativanone, medicarpin, and maackiain were purified from hydrolyzed extracts of *Ononis spinosa* root in our laboratory. The isoflavone glucoside standards (formononetin-, pseudobaptigenin-, onogenin-, sativanone-, maackiain-, and medicarpin glucoside) were isolated in our laboratory, too (Gampe et al., 2020). High-performance liquid chromatography (HPLC) and Mass spectrometry (MS)-grade methanol and acetonitrile were purchased from Fischer Scientific Co. (Fair Lawn, NJ, United States); LiChropur formic- and acetic acid were obtained from Merck (Darmstadt, Germany). Purified water was prepared using a Millipore Direct-Q system (Millipore Corp., Bedford, MA, United States).

### Plant Material

Transformed root cultures of *Ononis spinosa* L. and *Ononis arvensis* L. were obtained by the inoculation of sterile 6-week-old plants with *Agrobacterium rhizogenes* (strain R-1601). The segments of hypocotyls with fast-growing adventitious roots were transferred to Petri dishes containing MS medium (Murashige and Skoog, 1962), and cefotaxime was added to the medium (500 mg/l) for several subcultures until the total disappearance of *Agrobacterium* (Kuzovkina et al., 1996). After elimination of bacteria, the hairy roots were cultured in liquid Gamborg B5 (Gamborg et al., 1968) media in Erlenmeyer flasks, in a CERTOMAT BS-4 programmable incubation shaking cabinet (Braun Biotech International, Melsungen, Germany) at 100 rpm at 23 ± 2°C in the dark, and were subcultured every 21 days. Genomic DNA was extracted from hairy roots using the protocol and reagents of the Maxwell 16 LEV Plant DNA Kit (Madison, WI, United States). Polymerase chain reaction was executed for the confirmation of the presence of *rolB* rooting locus. The primers used to detect *rolB* were forward 5′-GAAGGTGCAAGCTACCTCTC-3′ and reverse 5′-GCTCTTGCAGTGCTAGATTT-3′ designed by Furner et al. (1986) (Bio Basic Canada Inc.). A PCR program described by Bertóti et al. (2019) was applied in a Bio-Rad iCycler machine (Hercules, CA, United States). Amplified PCR products were separated using electrophoresis on a 2% *w*/*v* agarose gel (Bio-Rad, CA, United States). From the two species, three parallel samples were inoculated (both 21-day-old, *O. spinosa* average mass 0.74 g and *O. arvensis* average mass 0.58 g) in 100-ml Erlenmeyer flasks with fresh liquid B5 medium (40 ml) and cultivated in the same shaking cabinet as before mentioned (100 rpm at 23 ± 2°C in the dark). Sampling of plant material was carried out at the time of inoculation (0 h) and at 7, 13, 21, and 28 days after inoculation.

### Sample Preparation

For the qualitative study, 0.10 g of freeze-dried (Christ Alpha 1-4 liophilizator, Braun, Melsungen, Germany) and ground hairy root culture was extracted with 5 ml 70% methanol using sonication for 30 min at room temperature. For quantitative analysis, 0.100 g powdered plant material was weighed and 50 μl of the internal standard (2.0 mg/ml naringenin solution) was added first, and then, the samples were extracted with 5 ml 70% methanol by sonication for 30 min. The samples were centrifuged, and the pellet was repeatedly extracted twice more with the same method. The collected supernatants were filled up to 25 ml, out of which 1 ml was filtered through a 0.22-μm PTFE filter (Nantong FilterBio Membrane Co., Ltd., Nantong, Jiangsu, China). From these, 200 μl were taken out and kept at 83°C for 5 h prior to HPLC analysis in order to hydrolyze malonate esters (Gampe et al., 2020). Investigating the liquid media for possible exudation of isoflavonoids, it was filtered and analyzed directly.

### UHPLC-ESI-Orbitrap-MS/MS Conditions for the Qualitative Analysis of Hairy Root Samples

For obtaining high-resolution mass spectrometric data of hairy root cultures, a Dionex UltiMate 3000 UHPLC system (3000RS diode array detector, TCC−3000RS column thermostat, HPG−3400RS pump, SRD−3400 solvent rack degasser, and WPS−3000TRS autosampler) was used hyphenated with a Orbitrap Q Exactive Focus Mass Spectrometer equipped with electrospray ionization (Thermo Fisher Scientific, Waltham, MA, United States). The UHPLC separation of the samples was attained on a Waters XSelect CSH Phenyl-Hexyl phase column (100 × 2.1 mm i.d.; 3.5 μm; Waters Corporation, Milford, MA, United States). Mobile phase consisted of 0.1% *v*/*v* formic acid (A) and 8:2 acetonitrile:0.1% *v*/*v* formic acid (B). The following gradient program was applied: 0 min, 20% B; 15 min, 80% B; 20 min, 80% B; and 22 min, 20% B. Solvent flow rate was 0.3 ml/min, and the column temperature was set to 25°C. The injection volume was 2 μl. The electrospray ionization source was operated in positive ionization mode, and operation parameters were optimized automatically using the built−in software. The working parameters were as follows: spray voltage, 3,500 V; capillary temperature, 256.25°C; sheath gas (N_2_), 47.5°C; auxiliary gas (N_2_), 11.25 arbitrary units; and spare gas (N_2_), 2.25 arbitrary units. The resolution of the full scan was of 70,000, and the scanning range was between 120 and 1,000 *m*/*z* units. The most intense ions detected in full scan spectrum were selected for MS/MS scan at a resolving power of 35,000, in the range of 50–1,000 *m*/*z* units. Parent ions were fragmented with normalized collision energy of 10%, 30%, and 45%.

### Isolation of Bulatlactone 2″-*O*-β-D-Glucoside and Ononilactone

Using an ultrasonic bath, 4.5 g of lyophilized, powdered sample of 4-week-old hairy root cultures of *O. spinosa* were extracted with 200 ml 50% methanol for 30 min. The extract was filtered and dried under reduced pressure at 60°C. The residue was redissolved in 5 ml 30% methanol and purified using the same flash chromatographic method mentioned at the isolation of standard compounds. The fractions eluting between 2 and 3 min were unified and further separated on a preparative HPLC system using eluents of 0.3% *v*/*v* acetic acid (A) and methanol (B). Gradient elution was used with the following program: 0 min, 30% B; 10 min, 30% B; and 20 min, 100% B with a 10-ml/min flow rate. Bulatlactone eluted at 7.5 min and the yield was 12.1 mg. From flash chromatography fractions eluted between 13 and 14 min, ononilactone was isolated using the same preparative HPLC system with the following eluents: 0.3% *v*/*v* acetic acid (A) and acetonitrile (B). The used gradient was as follows: 0 min 40% B up to 43% in 20 min. The peak eluted at 11.8 min was further purified using an isocratic method consisting of 63% methanol and 37% 0.3% *v*/*v* acetic acid with a 10-ml/min flow rate. The peak of interest eluted at 12.5 min and the yield was 1.4 mg.

### Nuclear Magnetic Resonance Spectroscopy

Nuclear magnetic resonance (NMR) spectral studies for ononilactone were carried out on Avance III HDX spectrometer from Bruker BioSpin GmbH (Rheinstetten, Germany): 800 MHz (equipped with a ^1^H&^19^F/^13^C/^15^N TCI CryoProbe ^1^H: 799.7 MHz, ^13^C: 201.0 MHz) and 500 MHz (with a 500 S2 ^1^H/^13^C/^15^N TCI Extended Temperature CryoProbe, ^1^H: 499.9 MHz, ^13^C: 125.7 MHz). Standard pulse sequences available in the TopSpin 3.5 pl 7 software were used for spectral acquisition, while the spectra were processed in MestreNova (Mestrelab Research). The complete resonance assignments were established from scalar and through-space ^1^H-^1^H, direct ^1^H-^13^C, and long−range ^1^H-^13^C connectivities on the basis of 1D ^1^H, ^13^C as well as 2D COSY, ROESY (CW spinlock for 250 ms), ^1^H-^13^C multiplicity-edited HSQC (^1^*J*_CH_ = 140 Hz), and ^1^H-^13^C HMBC (*^*n*^J*_CH_ = 7 and 2.5 Hz) spectra, respectively. The sample temperature was maintained at 298 K, and standard 5-mm NMR tubes were used. The ^1^H and ^13^C chemical shifts were referenced to the solvent signal of CHD_2_SOCD_3_ at δ_*H*_ = 2.500 ppm and the resonance line of CD_3_SOCD_3_ at δ_*C*_ = 39.520 ppm, respectively. The sample was dissolved in 600 μl DMSO-*d*_6_ (VWR International L.L.C.) and acidified by two drops of neat trifluoroacetic acid (TFA). NMR experiments for the structural analysis of bulatlactone 2″-*O*-β-D-glucoside were carried out in D_2_O on a 600-MHz Varian DDR NMR spectrometer (Agilent Technologies, Palo Alto, CA, United States) equipped with a 5-mm inverse-detection gradient (IDPFG) probehead. Standard pulse sequences and processing routines available in VnmrJ 3.2C/Chempack 5.1 were used for the structure identification. The complete resonance assignments were established from scalar and through-space ^1^H-^1^H, direct ^1^H-^13^C, and long−range ^1^H-^13^C connectivities as described above. The probe temperature was maintained at 298 K, and standard 5-mm NMR tubes were used. The ^1^H chemical shifts were referenced to the residual solvent signal δ_*H*_ = 4.790 ppm.

### Preparation of Stock Solutions, Calibration Standards, and Quality Control Samples

Individual stock solutions of the standards were prepared by dissolving the compounds in 70% methanol containing the internal standard (50 μl 2.0 mg/ml naringenin solution diluted to 25 ml) to obtain ∼1 mg/ml solutions. Equal parts of the standard solutions were mixed to gain the stock solution. Calibration standards were prepared by diluting the stock solution with the solution of the internal standard. The 10-point calibration curve was prepared using the following: 100, 60, 30, 10, 6, 3, 1, 0.6, 0.3, and 0.1 μg/ml concentration levels. QC samples were prepared separately from the stock solution at 50, 5, and 0.5 μg/ml nominal concentrations.

### UHPLC-UV-DAD Conditions for the Quantitative Analysis of *Ononis* Samples

Quantitative measurements were executed on a Waters ACQUITY UPLC system (sample manager, binary solvent manager, and PDA detector) (Waters Corporation, Milford, MA, United States). The samples were analyzed using the same phenyl-hexyl column as mentioned at the qualitative studies. Aiming at the determination of isoflavone derivatives, the same eluents were used, with the following gradient program: 0 min, 10% B; 15 min, 30% B; 17 min, 100% B; and 19 min, 10% B with 0.4 ml/min flow rate and 5 μl injected volume, and the column was heated to 40°C. For quantification of the isoflavanone and pterocarpan derivatives, the following gradient was used: 0 min, 25% B; 5 min, 25%; 6 min, 29% B; 15 min, 29%; 17 min, 100% B; and 19 min, 25% B with 0.4 ml/min flow rate and 5 μl injected volume, and the column was heated to 27°C (Gampe et al., 2020).

## Results

### Characterization of Hairy Root Growth

**FIGURE 1:**
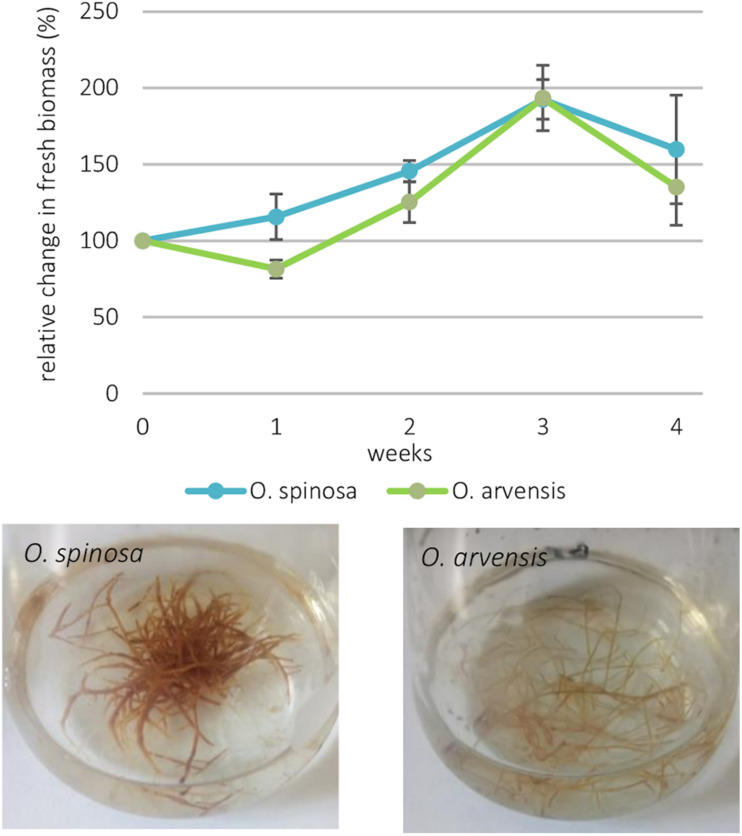
The relative change in the two cultures’ biomass production with their standard deviation (*n* = 3) and the cultures after 21 days.

**FIGURE 2:**
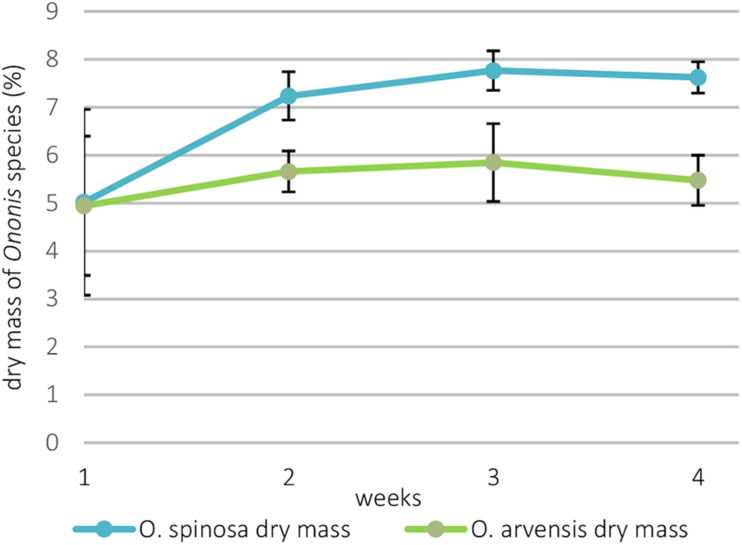
The dry mass of the cultures expressed as mass percentage with their standard deviation (*n* = 3).

For both *Ononis* species, the complete and stable transformation status of the isolated hairy root cultures (HRCs) was confirmed by PCR on their genomic DNA for the detection of the presence of the proto-oncogene *rolB*. In general, the HRCs of *O. spinosa* showed a more robust phenotype and darker color, than *O. arvensis* (Figure 1). Investigating the change in biomass, a very similar trend could be observed. The biomass of both species increased until the 3rd week. In the first 2 weeks, the increase was lighter in the case of *O. arvensis*. During the 3rd week, both cultures reached their maximum in biomass production and their growth did not differ significantly. Reaching the 4th week, the biomass started to decrease, showing the aging of the cultures. The dry masses showed a similar pattern; however, *O. spinosa* cultures showed a significantly higher dry mass. *O. arvensis* HRCs followed a similar trend, but the change in the dry mass is not significant (Figure 2).

### Qualitative Characterization of Phytochemical Composition of *Ononis* HRCs

**FIGURE 3:**
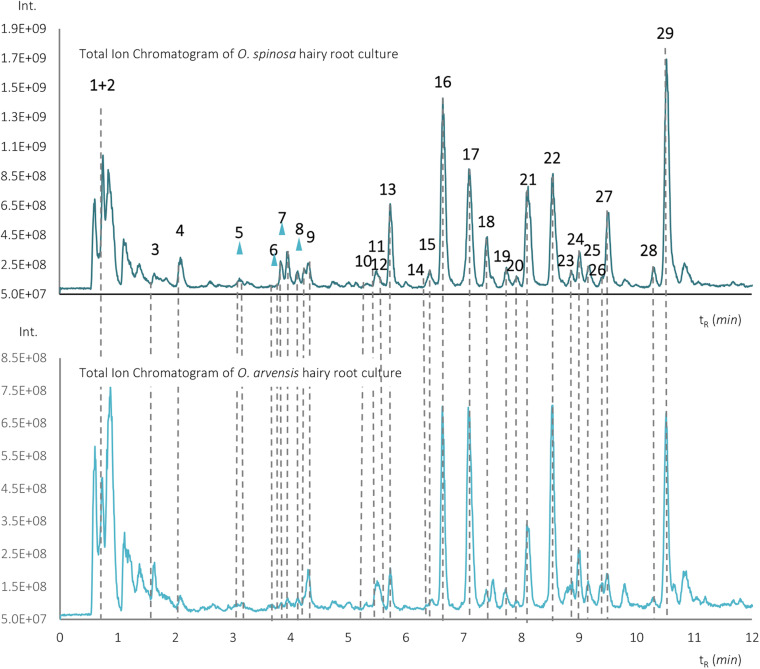
Total ion chromatograms of the aqueous-methanolic extracts of *O. spinosa* and *O. arvensis* hairy root cultures.

The total ion chromatogram (TIC) recorded in positive ionization mode of the 70% aqueous-methanol extracts with the identified compounds of *O. spinosa* and *O. arvensis* HRCs can be seen in Figure 3.

The identified compounds along with their number, retention time, protonated pseudo-molecular ions, and most intense product ions are shown in Supplementary Table 1. All identified compounds could be observed in both species.

The most characteristic peaks of the samples were isoflavonoid derivatives. Isoflavonoid derivatives were identified following the methods described in our previous publication (Gampe et al., 2016, 2018a,b). In the HRCs, the isoflavonoid derivatives could be found in the forms of glucosides (10, 12, 13, 14, 16, and 17), glucoside malonates (17, 20, 21, and 22), aglycones (24, 25, 27, 28, and 29), and homopipecolic acid esters of glucosides (5, 6, 7, and 8) (Supplementary Table 1). Based on solely HR-MS/MS studies, the type of hexoside and the position of the malonate moiety could not be deduced, so the compounds were identified tentatively as 7-*O*-glucosides and 7-*O*-glucoside 6″-*O*-glucoside malonates based on the works of Farag et al. (2007) and de Rijke et al. (2004). Because of the racemic feature of the beta amino acid moiety, the homopipecolic acid derivatives can be found in the form of diastereomeric pairs separated to double peaks on the stationary phase (Supplementary Table 1). The first two peaks were identified as the methyl-esters of homoproline and homopipecolic acid; the esterification of homoproline in methanol as extraction solvent has already been reported before (Paßreiter, 1992). Although homoproline could be found in the form of methyl ester, the corresponding homoproline isoflavonoid glucoside esters could not be detected.

**FIGURE 4:**
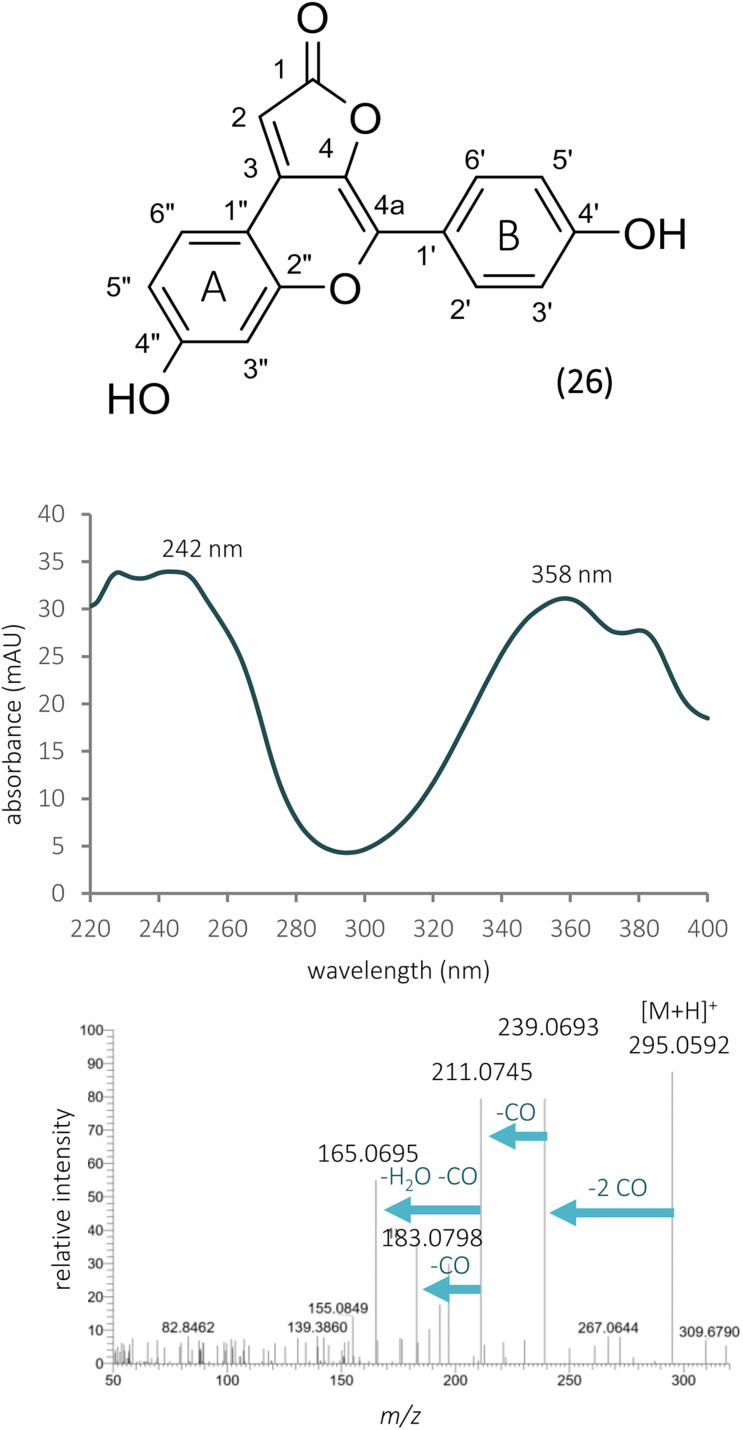
The structure, UV, and MS/MS spectra of ononilactone.

Beside isoflavonoid derivatives, special phenolic lactones (norneolignans) were detected in the forms of glucosides (9 and 11) and aglycones (15 and 23). These derivatives of puerol A and clitorienolactone B were known in *Ononis* species before (Ghribi et al., 2015; Addotey et al., 2018; Gampe et al., 2018b). Surprisingly, a compound (peak 26) with a different fragmentation profile and a rather unusual UV spectrum with two absorption maxima was also observed eluting with the aglycones (Figure 4).

The solution of this compound in aqueous-polar organic solvents and DMSO showed a strong blue fluorescence if irradiated with UV light of 366 nm. The most dominant fragment ions originated from the sequential losses of CO and H_2_O units. Based on HR-MS analysis, the observed protonated pseudo-molecular ion showed an *m*/*z* 295.0591 value, and the calculated formula was C_17_H_10_O_5_, suggesting the presence of 13 double bond equivalents. Since broadened aromatic ^1^H NMR signals could be observed in DMSO-*d*_*6*_, hindering the observation of key ^1^H-^13^C correlations in the heteronuclear 2D spectra, two drops of TFA was applied to overcome line broadening. NMR spectra of the acidified spectrum enabled the structure elucidation of compound 26 from only 49 μM material (ca. 9 μg in the NMR tube). In the ^13^C NMR spectrum, the presence of a carbonyl group at δ_*C*_ 170.5; six quaternary carbons at δ_*C*_ 163.2, 160.0, 146.4, 135.4, 134.1, 120.0, and 107.4; one methine carbon at δ_*C*_ 89.1; and nine aromatic methine carbons between δ_*C*_ 103.0 and 129.8 were confirmed (Table 1). The ^1^H NMR contained spectrum seven aromatic and one methine proton (see Table 1).

**TABLE 1:** ^1^H, ^13^C, and 2D NMR data of ononilactone in DMSO-d_6_ acidified by TFA [δ (ppm), *J* (Hz)].

**No.**	**^1^H**	**^13^C**	**HMBC**
**1**	–	170.5	
**2**	6.07 s	89.1	170.5, 146.4, 107.4
**3**	–	146.4	
**4**	–	134.1	
**4a**	–	135.4	
**1**′	–	120.0	
**2**′	8.01 d *J* = 8.4	129.0	129.0, 135.4, 160.0
**3**′	7.00 d *J* = 8.4	116.2	120.0, 116.2, 160.0
**4**′	–	160.0	
**5**′	7.00 d *J* = 8.4	116.2	120.0, 116.2, 160.0
**6**′	8.01 d *J* = 8.4	129.0	129.0, 135.4, 160.0
**1**″	–	107.4	
**2**″	–	152.7	
**3**″	7.02 d *J* = 2.3	103.0	107.4, 115.4, 153.0, 161.8
**4**″	–	161.8	
**5**″	6.98 dd *J* = 8.5, 2.3	115.4	107.4, 103.0
**6**″	7.94 d *J* = 8.5	128.0	146.4, 152.7, 163.2

**FIGURE 5:**
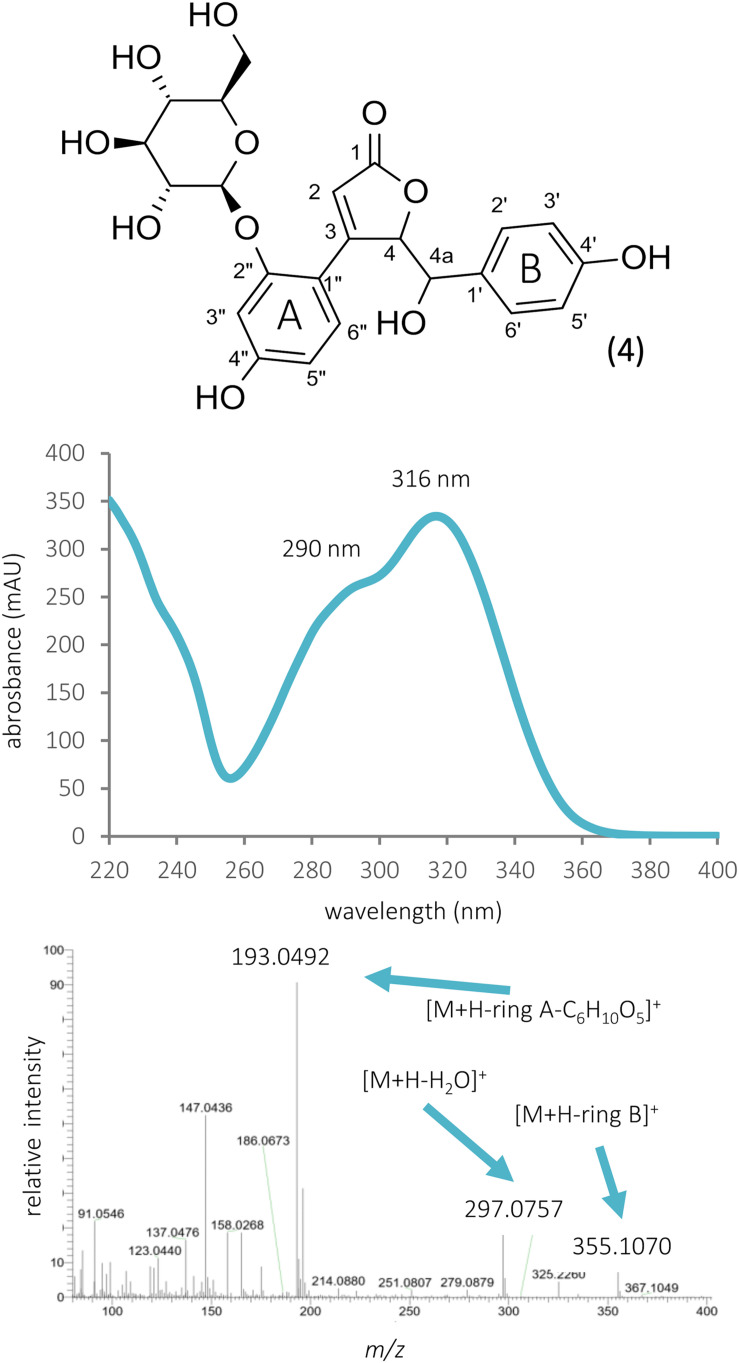
The structure, UV, and MS/MS spectra of bulatlactone 2″-*O*-β-D-glucoside.

Investigating the early eluting peaks, peak 4 showed a UV spectrum very similar to that of chlorogenic acid or other caffeoyl acid derivatives (Figure 5). Moreover, in the HR-MS spectrum, a peak at *m*/*z* 355.1054 could be detected, which could result in the same molecular formula (C_16_H_18_O_9_) as chlorogenic acid, but the fragmentation profile did not match. The ^1^H NMR spectrum revealed glucose resonances (δ_*H*_ 3.54, 3.62, 3.70, 3.70, 3.80, 3.98, and 5.20) and two aromatic spin systems. Similarly to compound 26, a *para*-disubstituted phenyl ring gives the resonances at δ_*H*_ 6.71 and 6.87 ppm, while the multiplets at δ_*H*_ 6.67, 6.79, and 7.25 indicate a 1,2,4 trisubstituted phenyl ring (Table 2). Three singlet signals were also recorded at δ_*H*_ 5.19, 6.07, and 6.22. The lack of scalar coupling between the adjacent methine protons may be the consequence of their ca. 90 dihedral angle, according to the Karplus relationship.

**TABLE 2:** ^1^H, ^13^C, and 2D NMR data of bulatlactone 2″-*O*-β-D-glucoside in D_2_O [δ (ppm), *J* (Hz)].

**No.**	**^1^H**	**^13^C**	**HMBC**
**1**	–	177.0	
**2**	6.07 s	113.9	87.1, 111.8, 164.1, 176.9
**3**	–	164.1	
**4**	6.22 s	87.1	73.4, 113.9, 127.8, 164.1, 176.9
**4a**	5.19 s	73.4	127.8, 128.6
**1**′	–	127.8	
**2**′	6.87 d *J* = 8.4	128.6	73.4, 114.5, 128.6, 155.4
**3**′	6.71 d *J* = 8.4	114.5	127.8, 114.5, 155.4
**4**′	–	155.4	
**5**′	6.71 d *J* = 8.4	114.5	127.8, 114.5, 155.4
**6**′	6.87 d *J* = 8.4	128.6	73.4, 114.5, 128.6, 155.4
**1**″	–	111.8	
**2**″	–	156.1	
**3**″	6.79 d *J* = 1.7	102.5	111.8, 156.1, 160.5
**4**″	–	160.5	
**5**″	6.67 dd *J* = 8.5, 1.7	110.5	102.5, 111.8
**6**″	7.25 d *J* = 8.5	131.8	156.1, 160.5, 164.1
**G1**	5.20 d *J* = 9.4	99.6	156.1
**G2**	3.70 t *J* = 9.4	72.7	
**G3**	3.62 t *J* = 9.4	75.8	
**G4**	3.54 t *J* = 9.4	69.2	
**G5**	3.70 t *J* = 9.4	76.2	
**G6a**	3.80 d *J* = 12.0	60.5	
**G6b**	3.98 dd *J* = 12.0, 5.7		

### Quantitative Analysis of Isoflavonoids in *Ononis* HRCs

**FIGURE 6:**
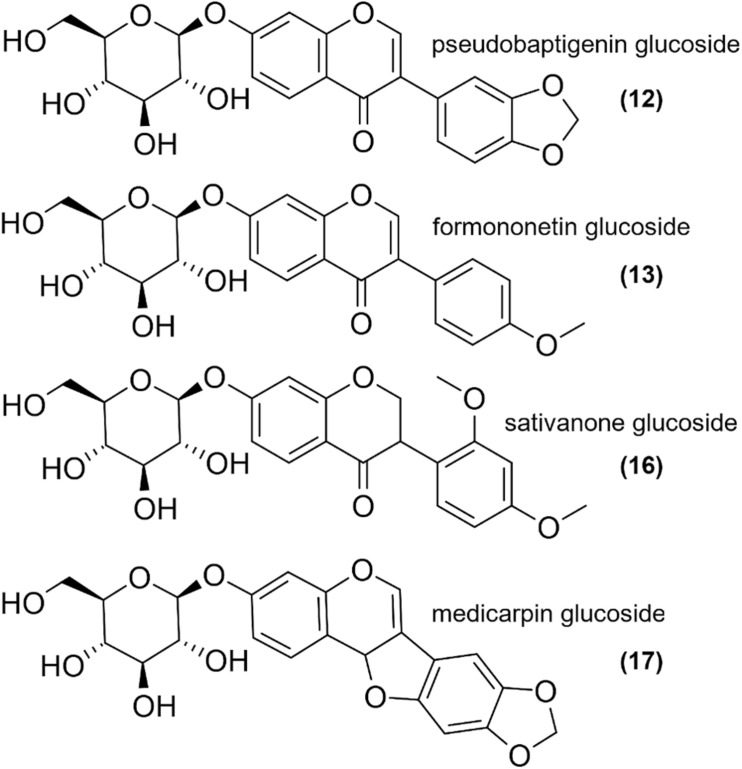
The most abundant structures found in *Ononis* HRC extracts.

**FIGURE 7:**
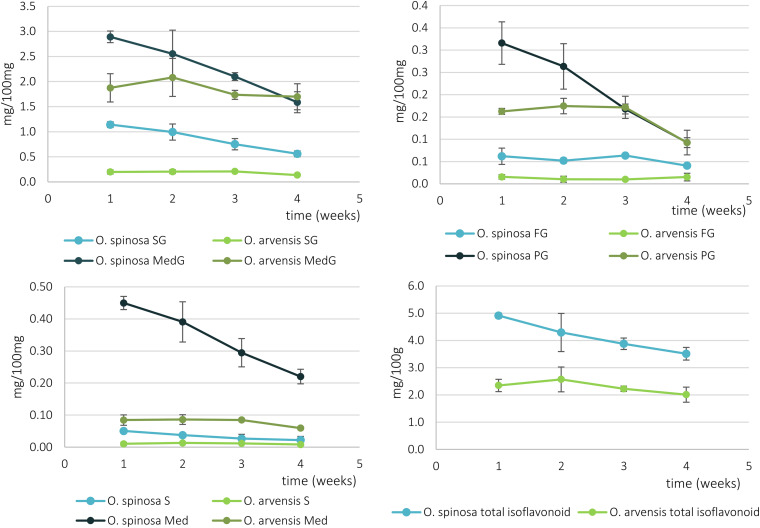
The relative isoflavonoid content of *O. spinosa* and *O. arvensis* HRCs in mg/100 mg (SG, sativanone glucoside; MedG, medicarpin glucoside; S, sativanone; Med, medicarpin; PG, pseudobaptigenin glucoside; FG, formononetin glucoside).

**FIGURE 8:**
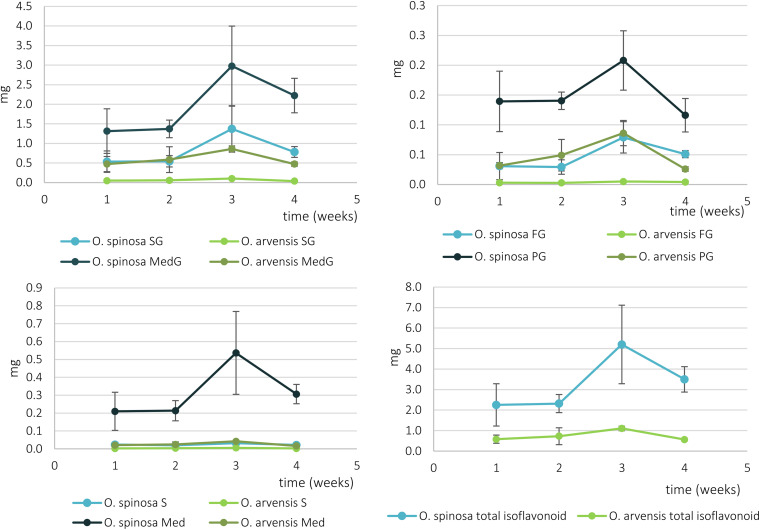
The absolute isoflavonoid content of *O. spinosa* and *O. arvensis* HRCs in milligrams (SG, sativanone glucoside; MedG, medicarpin glucoside; S, sativanone; Med, medicarpin; PG, pseudobaptigenin glucoside; FG, formononetin glucoside).

Using the same UHPLC-UV-DAD method developed for the characterization of wild-grown *Ononis* species, the relative and absolute isoflavonoid contents of HRCs were evaluated (Supplementary Tables 2, 3). Firstly, the liquid media were investigated, but no isoflavonoid derivatives could be detected. In the *in vitro* cultures, the main compounds were sativanone glucoside and medicarpin glucoside followed by pseudobaptigenin glucoside and formononetin glucoside (Figure 6). The aglycones could be observed in a magnitude lower quantity for sativanone and medicarpin, whereas in the case of isoflavones, they were under limit of detection (Supplementary Tables 2, 3). In *O. spinosa* samples, the isoflavonoid concentration (mg/100 mg) showed a constant regression from the 1st week, whereas in *O. arvensis* samples, up to the 2nd week the level increased, then dropped (Figure 7). The *O. spinosa* cultures possessed a higher level of total isoflavonoid content than *O. arvensis* (Figures 7, 8). If the absolute quantities of isoflavonoids were investigated (Figure 8), without the correction of the biomass, both samples reached their maximum at 3rd week (similarly to the biomass). In the 4th week, the isoflavonoid levels dropped, indicating their breakdown or transformation (Figure 8).

## Discussion

### Structural Identification of the New Compounds

The UV spectrum of compound 26 resembled to that of flavonoids; however, the bands did not completely overlap with that of standard compounds (Markham and Mabry, 1975). The MS/MS fragmentation spectrum recorded in positive ionization mode did not show any specific patterns characteristic for flavonoids or isoflavonoids (Cuyckens and Claeys, 2004). The presence of two phenyl rings with *para* (ring B) and *ortho-para* (ring A) substitution patterns could be deduced from the coupling constants of ^1^H resonances and ^1^H-^1^H COSY experiments. Based on the ^1^H NMR chemical shifts and 2D HMBC correlation peaks, the protons at δ_*H*_ 8.01 were shown to belong to H-2′ and 6′, while protons at δ_*H*_ 7.00 to H-3′ and 5′ of ring B, respectively. The coupling pattern of the ^1^H resonances at δ_*H*_ 7.94, 7.02, and 6.98 assigns these chemical shifts to H-3″, H-6″, and H-5″ (ring A), respectively. These aromatic protons served as good entry points to assign the quaternary carbon atoms of ring B. Using the HMBC data, the C-1″, C-2″, and C-4″ atoms were assigned to peaks at δ_*C*_ 107.4, δ_*C*_ 152.7, and δ_*C*_ 163.2 ppm, respectively. The sharp singlet at δ_*H*_ 6.07 showed a HMBC correlation peak to C-1″, indicating a connection of a –C= CH– unit to ring A. The position of the H-2 proton has also been confirmed by the ROESY crosspeak of H-6″ and H-2. The same proton (δ_*H*_ 6.07) showed a HMBC crosspeak to the carbonyl δ_*C*_ 170.5, which permitted the deduction that this olefinic group is linked to an ester or lactone. The structural motif, which could be drawn up regarding the information gained from the NMR experiments, showed a close similarity with the structure of puerol A (Ghribi et al., 2015), so that the presence of a δ-lactone was presumed, which could be confirmed by the HMBC correlations of H-2 with both C-3 and C-4. In the case of puerol derivatives or clitorienolactones (Addotey et al., 2018; Gampe et al., 2018b), one of the phenolic rings (ring A) is linked to the lactone ring through a methylene group. However, in our case, a CH_2_ unit could not be observed, and the other phenolic ring (ring B) showed a linkage through a quaternary carbon atom based on HMBC experiments. Regarding the molecular formula and the structures of puerol derivatives, an ether bridge between C-2″ and C-4a was hypothesized. With this linkage, a furanoflavonoid-like structure is formed (Figure 4), which can explain the similarity of the UV spectrum and the blue fluorescence. As this compound is described for the first time, the name ononilactone was chosen for this new skeleton.

**FIGURE 9:**
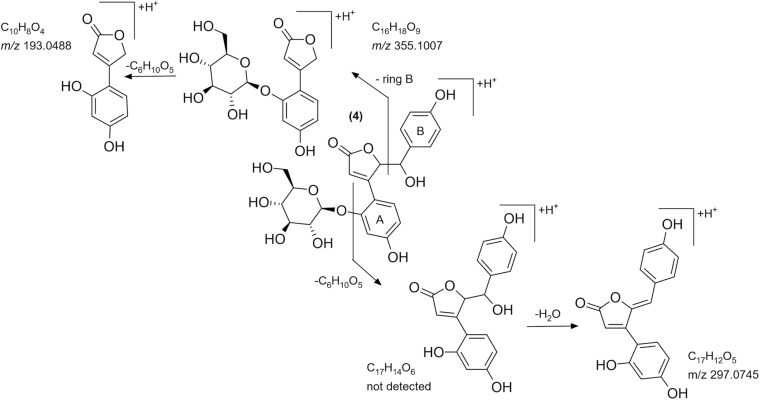
The tentative MS/MS fragmentation pathway of bulatlactone 2″-*O*-β-D-glucoside.

Although the UV spectrum, HR-MS base peak, and the calculated formula strongly resembled to chlorogenic acid, looking at the MS/MS spectrum of compound 4, the most intense fragment rose at *m*/*z* 193.0491, which was in disagreement with the MS/MS data of chlorogenic acid registered by other research groups [Metabocard of Chlorogenic Acid (HMDB0003164), 2006]. The mass difference (162 Da) between the two peaks (Figure 5) led us to the assumption that this compound is a glycoside, which could lose a hexose unit as a neutral loss. With the same exact mass and molecular formula, scopolin is mentioned in the literature, as a glycosidic compound. Furthermore, scopolin and its aglycone scopoletin were isolated from *O. arvensis* (Sichinava et al., 2014). Nevertheless, the UV spectrum of these compounds shows an absorption maximum at higher values (Pina et al., 2019), and the fragmentation pathway does not match with that of coumarins (Yi et al., 2014). As a consequence, the isolation of the compound was inevitable to elucidate its structure by NMR spectroscopy. The number of carbon and hydrogen resonances were not in agreement with that of the hypothesized one by the HR-MS measurements (C_16_H_18_O_9_), but the presence of a glucose unit could be confirmed. The recorded NMR signals showed great similarity with that of puerol A (Ghribi et al., 2015), except for the lack of a CH_2_ signal at 4a position. The downfield shifts of H-4a and C-4a indicated the presence of a hydroxy group in geminal position (Figure 5). Regarding the structure of the aglycone and the glucoside drawn from the NMR studies, the molecular formulas C_17_H_14_O_6_ and C_23_H_24_O_11_ could be calculated, resulting in calculated protonated quasi-molecular masses of 315.0863 and 477.1391. Revising the HR-MS spectrum of peak 4, none of these signals could be detected; however, the [M + Na]^+^, [2M + H]^+^, and [2M + Na]^+^ ions were present at *m*/*z* 499.1224, 953.7456, and 975.6811, respectively. Instead of the aglycone as a product ion, the [M + H-ring B]^+^ ion could be detected at *m*/*z* 355.1054, which indeed could lose a glucose moiety resulting in the peak at *m*/*z* 193.0491 (Figure 9). Neither the aglycone nor the glucoside form of this compound have been described before in the plant kingdom; thus, it is named as bulatlactone 2″-*O*-β-D-glucoside.

**FIGURE 10:**
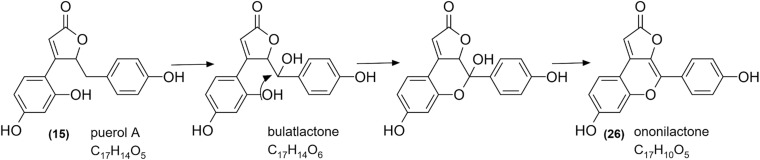
The putative synthetic pathway of ononilactone.

The presence and structure of bulatlactone is fascinating from the point of view that it can serve as an intermediate between the well-known puerol derivatives and the newly described structure, ononilactone (Figure 10). As we assume, through a dehydration and a ring closure step, ononilactone could be formed from bulatlactone aglycone. Bulatlactone was only detected in the form of 2″-*O*-glucoside and not as an aglycone. As the glucosidation takes place through the 2″ hydroxy group (which is involved in the ring closure, as well), it prevents the transformation of bulatlactone to ononilactone and stabilizes this form.

### Qualitative Characterization of Phytochemical Composition of *Ononis* HRCs

Investigating the isoflavonoid pattern of the HRCs of the species, a reduced spectrum could be observed compared to the native plants. Formononetin, 2′-methoxyformononetin, sativanone, and medicarpin beside their derivatives could be found in both wild-grown (Ghribi et al., 2015) and genetically modified transformed samples abundantly. On the contrary, isoflavonoid derivatives with various skeletons (isoflavone, isoflavanone, and pterocarpan) but with a common methylenedioxy substituent (pseudobaptigenin, cuneatin, onogenin, and maackiain) could only be detected in the HRCs in trace quantities or not at all. Interestingly, pseudobaptigenin derivatives could be observed only in trace quantities in the samples for qualitative analysis; however, in the quantitative samples, their concentration was comparable with formononetin. This could be the consequence of that after lyophilization, quantitative samples were immediately measured, while the qualitative samples were kept airtight and analyzed only weeks after the harvest. The decomposition of pseudobaptigenin in aqueous medium was experienced by our research group during *in vitro* tests, but in this case, the HRC were kept sealed in a dry form. Moreover, in the qualitative samples, onogenin and maackiain derivatives could be observed, while in quantitative samples, their amounts were under limit of detection. Based on these observations, it is hypothesized that pseudobaptigenin is transformed to onogenin and maackiain. These results show that the measured isoflavonoid content of wild grown samples (Gampe et al., 2020) does not necessarily reflect the isoflavonoid content of the living plant, as it changes with time after harvest. As different isoflavonoids can possess distinct biological effects, the age and the storage conditions can affect the medicinal value of the sample.

### Proliferation of HRCs and Comparison of Biomass and Phenolic Compound Accumulation

The overall isoflavonoid yield of HRCs showed a somewhat higher level, than the wild-grown samples of *O. spinosa*, and was comparable of that of *O. arvensis*. However, the aim of this study was not to optimize the proliferation and isoflavonoid extractability, thus modifying the circumstances of cultivation can lead to better results. Moreover, the isoflavonoid profile of wild-grown samples markedly differed from the fresh and older samples, too (Gampe et al., 2020). The most characteristic compound produced by the HRCs was medicarpin glucoside (2.23–2.89 mg/100 mg in *O. spinosa* and 1.69–1.87 mg/100 mg in *O. arvensis*), followed by sativanone glucoside (0.56–1.14 mg/100 mg in *O. spinosa* and 0.13–0.20 mg/100 mg in *O. arvensis*) and pseudobaptigenin glucoside (0.12–0.20 mg/100 mg in *O. spinosa* and 0.09–0.16 mg/100 mg in *O. arvensis*) (Supplementary Tables 2, 3). In the wild-grown samples, the amount of methoxy and methylenedioxy derivatives are comparable, whereas in HRCs, the methoxy derivatives are the predominant, except for pseudobaptigenin. The observed decrease in the amount of the methylenedioxy compounds could be a result of the genetic modification by the Ri plasmid or the lack of some biotic or abiotic factors that could not be reproduced under *in vitro* circumstances, e.g., symbiotic *Rhizobium* strains and drought stress. Regarding the relative isoflavonoid content that decreased from the 1st (*O. spinosa*) or the 2nd week (*O. spinosa*), but the fresh and dry weight increased until the 3rd week, it can be assumed that the cultures use their sources mainly for growth and not accumulating specialized metabolites. If the aim is the isolation of isoflavonoid compounds, the 3rd week is optimal, since the absolute quantity of the isoflavonoids was the highest in those days. Usually, the aglycone forms are regarded as the biologically active forms, but unfortunately, fresh HRCs accumulate mainly glycosides. On the other hand, regarding the qualitative studies, upon storage, these can transform to aglycones or the intestinal flora can hydrolyze them to their aglycone form, too.

In conclusion, *Ononis* hairy root cultures contain some special phenolic lactones beside isoflavonoids. *O. spinosa* can serve as rich sources of methoxylated isoflavonoids, as it produced them in higher quantities compared to wild-grown plants. Considering that most isoflavonoids with methylenedioxy substituent are missing, the isoflavonoid spectrum of HRCs is less complicated, providing an easy possibility to realize the isolation of the present compounds.

## Data Availability Statement

The original contributions presented in the study are included in the article/Supplementary Material, further inquiries can be directed to the corresponding author.

## Author Contributions

ZS recorded and analyzed the NMR spectra for ononilactone and AD recorded the ones for bulatlactone. IB recorded the Orbitrap-MS chromatograms. IK and ÉS started and maintained the hairy root cultures. LK and SB contributed to the design of the experiments and assisted in the interpretation of the data. NG performed all other experiments, analyzed and interpreted the data, and drafted the manuscript with the assistance of SB. All authors read and approved the final version of the manuscript.

## Conflict of Interest

ZS was employed by the company Gedeon Richter Plc. The remaining authors declare that the research was conducted in the absence of any commercial or financial relationships that could be construed as a potential conflict of interest.
