# Supplementary material for: Qualitative and Quantitative Phytochemical Analysis of Ononis Hairy Root Cultures
Source: Front Plant Sci. 2021 Jan 13;11:622585. doi: 10.3389/fpls.2020.622585 (PMC7874045; doi:10.3389/fpls.2020.622585)
Supplement: Supplementary file 1 [file Data_Sheet_1.docx]

Supplementary Material

# Qualitative characterization of phytochemical composition of *Ononis* HRCs

## Table S1: The identified compounds and their high-resolution MS and MS/MS data of *O. spinosa* and *O. arvensis* hairy root culture extracts

| **No** | **t_R_** | **[M+H]^+^** | **delta** | **Formula** | **Aglycone** | **MS/MS fragment ions** | **Identification** |
| --- | --- | --- | --- | --- | --- | --- | --- |
|  | *min* | *m/z* | *ppm* |  | *m/z* | *m/z* |  |
| 1 | 0.74 | 144.1015 | -0.52 | C_7_H_13_NO_2_ |  | 70.065 | Methyl-ester of homoproline (artifact) |
| 2 | 0.74 | 158.1172 | -0.42 | C_8_H_15_NO_2_ |  | 84.0812 | Methyl-ester of homopipecolic acid (artifact) |
| 3 | 1.64 | 433.1338 | -0.58 | C_18_H_24_O_12_ |  | 145.0493, 127.0390 | Licoagroside B |
| 4 | 2.11 | 477.1375 | -1.63 | C_23_H_25_O_11_ | 315.0853 | 355.1007, 297.0745, 193.0488, 165.0541, 147.0435 | Bulatlactone 2”-*O*- β-D-glucoside |
| 5 | 3.09, 3.25 | 556.2177 | -0.04 | c_29_h_33_no_10_ | 269.0801 | 288.1436, 144.1017, 84.0814 | Formononetin 7-*O*-β-D-glucoside 6"-piperidine 2-acetate |
| 6 | 3.67, 3.78 | 602.2231 | -0.17 | c_30_h_35_no_12_ | 315.0855 | 288.1435, 177.0543, 163.0387, 144.1017, 135.0439, 84.0814 | Onogenin 7-*O*-β-D-glucoside 6"-piperidine 2-acetate |
| 7 | 3.84, 3.95 | 588.2434 | -0.91 | c_30_h_37_no_11_ | 301.1064 | 288.1436, 273.1115, 163.0387, 144.1017, 135.0439, 84.0814 | Sativanone 7-*O*-β-D-glucoside 6"-piperidine 2-acetate |
| 8 | 4.12, 4.25 | 558.2326 | -1.38 | c_29_h_35_no_10_ | 271.0959 | 288.1435, 161.0594, 144.1017, 137.0595, 123.0441, 84.0814 | Medicarpin 3-*O*-β-D-glucoside 6"-piperidine 2-acetate |
| 9 | 4.32 | 461.1435 | -1.57 | c_23_h_24_o_10_ | 299.0908 | 281.0802, 253.0854, 239.0698, 193.0493, 107.0495 | Puerol A 2”-*O*- β-D-glucoside |
| 10 | 5.48 | 461.1426 | -2.47 | c_23_h_24_o_10_ | 299.0900 | 284.0666, 269.0796, 252.0405, 243.1005, 148.0513, 137.0592 | 2'-Methoxy formononetin 7-*O*-β-D-glucoside |
| 11 | 5.57 | 475.1593 | -1.21 | c_24_h_26_o_10_ | 313.1069 | 295.0960, 267.1012, 253.0855, 207.0647, 107.0495 | Clitorienolactone B 2”-*O*-glucoside |
| 12 | 5.64 | 445.1118 | -2.53 | c_22_h_20_o_10_ | 283.0590 | 253.0485, 225.0438, 196.1689 | Pseudobaptigenin 7-*O*-β-D-glucoside |
| 13 | 5.73 | 431.1345 | 1.95 | c_22_h_22_o_9_ | 269.0805 | 254.0570, 237.0543, 226.0622, 213.0907, 118.0415 | Formononetin 7-*O*-β-D-glucoside |
| 14 | 6.42 | 477.1382 | -1.97 | c_23_h_24_o_11_ | 315.0858 | 297.0753, 287.0909, 257.0805, 229.0857, 178.0623, 163.0388, 147.0439,  135.0440 | Onogenin 7-*O*-β-D-glucoside |
| 15 | 6.46 | 299.0909 | -1.67 | c_17_h_14_o_5_ |  | 281.0804, 253.0805, 239.0699, 193.0493, 107.0495 | Puerol A |
| 16 | 6.63 | 463.1591 | -1.67 | c_23_h_26_o_10_ | 301.1065 | 283.1271, 273.1116, 177.1119, 163.0388, 135.0440 | Sativanone 7-*O*-β-D-glucoside |
| 17 | 7.08 | 433.1487 | -1.41 | c_22_h_24_o_9_ | 271.096 | 137.0596 | Medicarpin 3-*O*-β-D-glucoside |
| 18 | 7.39 | 517.1353 | 2.42 | c_25_h_24_o_12_ | 269.0803 | 254.0569, 237.0541, 213.0906 | Formononetin 7-*O*-β-D-glucoside 6"-malonate |
| 19 | 7.51 | 531.1760 | -1.18 | c_25_h_22_o_113_ | 283.0597 | 253.0491, 225.0543, 197.0595, 169.0647 | Pseudobaptigenin 7-*O*-β-D-glucoside 6"-malonate |
| 20 | 7.92 | 563.1382 | -2.37 | c_26_h_26_o_14_ | 315.0855 | 297.0752, 287.0912, 257.0803, 229.0856, 178.0628, 163.0387, 147.0438,  135.0439 | Onogenin 7-*O*-β-D-glucoside 6"-malonate |
| 21 | 8.11 | 549.1595 | -1.4 | c_26_h_28_o_13_ | 301.1066 | 283.1001, 273.1110, 177.1144, 163.0471, 135.0455 | Sativanone 7-*O*-β-D-glucoside 6"-malonate |
| 22 | 8.53 | 519.1488 | -1.74 | c_25_h_26_o_12_ | 271.0959 | 161.0959, 137.0595, 123.0441 | Medicarpin 3-*O*-β-D-glucoside 6"-malonate |
| 23 | 8.9 | 313.1066 | -2.11 | c_18_h_16_o_5_ |  | 295.0961, 267.1012, 253.0856, 207.0650, 107.0495 | Clitorienolactone B |
| 24 | 8.99 | 271.0959 | 1.43 | c_16_h_14_o_4_ |  | 161.0595, 137.0595, 123.0441 | Medicarpin |
| 25 | 9.16 | 299.0916 | 0.67 | c_17_h_14_o_5_ |  | 284.0658, 267.0649, 252.0412, 243.1014, 213.0551, 163.0387, 148.0517,  137.0596 | 2'-methoxy formononetin |
| 26 | 9.39 | 295.0591 | -0.96 | C_17_H_10_O_5_ |  | 295.0644, 239.0693, 211.0745, 193.0589, 183.0798, 165.0695 | Ononilactone |
| 27 | 9.5 | 269.0803 | -1.99 | c_16_h_12_o_4_ |  | 269.0803, 253.0490, 237.0541, 226.0623, 225.0542, 213.0906, 197.0594 | Formononetin |
| 28 | 10.3 | 315.0858 | -1.63 | c_17_h_14_o_6_ |  | 297.0753, 287.0903, 257.0804, 229.0857, 178.0623, 163.0388, 147.0435,  135.0440 | Onogenin |
| 29 | 10.51 | 301.1061 | -3.16 | c_17_h_16_o_5_ |  | 273.1116, 177.1272, 163.0388, 1510388, 135.0440, 107.0495 | Sativanone |

## The NMR spectra of ononilactone

Figure S1: Partial ^1^H-NMR spectrum of ononilactone (800 MHz, DMSO-*d6* and TFA)

Figure S2: Partial ^13^C-NMR spectrum of ononilactone (500 MHz, DMSO-*d*6 and TFA)

Figure S3: Partial ^1^H-^1^H COSY spectrum of ononilactone (800 MHz, DMSO-*d*6 and TFA)

Figure S4: Partial ^1^H-^13^C HSQC spectrum of ononilactone (500 MHz, DMSO-*d*6 and TFA)

Figure S5: Partial ^1^H-^13^C HMBC spectrum of ononilactone (500 MHz, DMSO-*d*6 and TFA)

Figure S6: Partial ^1^H-^1^H ROESY spectrum of ononilactone (800 MHz, DMSO-*d*6 and TFA)

## The NMR spectra of bulatlactone 2”-*O*-glucoside

Figure S7: Partial ^1^H-NMR spectrum of bulatlactone 2”-*O*-glucoside (600 MHz, D_2_O)

Figure S8: Partial ^13^C-NMR spectrum of bulatlactone 2”-*O*-glucoside (600 MHz, D_2_O)

Figure S9: Partial ^1^H-^1^H COSY spectrum of bulatlactone 2”-*O*-glucoside (600 MHz, D_2_O)

Figure S10: Partial 1H-13C HSQC spectrum of bulatlactone 2”-*O*-glucoside (600 MHz, D2O)

Figure S11: Partial ^1^H-^13^C HMBC spectrum of bulatlactone 2”-*O*-glucoside (600 MHz, D_2_O)

Figure S12: Partial ^1^H-^1^H NOESY spectrum of bulatlactone 2”-*O*-glucoside (600 MHz, D_2_O)

# Quantitative analysis of isoflavonoids in *Ononis* HRCs

## Table S2: The mean relative isoflavonoid content of *O. spinosa* and *O. arvensis* HRC extracts in mg/100mg (n=3)

|  | Pseudobaptigenin glucoside  (*mg/100mg*) | Formononetin glucoside  (*mg/100mg*) | Sativanone glucoside  (*mg/100mg*) | Medicarpin glucoside (*mg/100mg*) | Sativanone (*mg/100mg*) | Medicarpine  (*mg/100mg*) | Total  (*mg/100mg*) |
| --- | --- | --- | --- | --- | --- | --- | --- |
| *O. spinosa* | | | | | | | |
| 1^st^ week | 0.31±0.05 | 0.06±0.02 | 1.14±0.06 | 2.89±0.12 | 0.050±0.004 | 0.45±0.02 | 4.91±0.10 |
| 2^nd^ week | 0.26±0.05 | 0.05±0.01 | 0.99±0.16 | 2.55±0.47 | 0.038±0.007 | 0.39±0.06 | 4.29±0.70 |
| 3^rd^ week | 0.16±0.01 | 0.059±0.001 | 0.75±0.11 | 2.23±0.08 | 0.027±0.013 | 0.39±0.04 | 3.87±0.21 |
| 4^th^ week | 0.12±0.03 | 0.051±0.002 | 0.56±0.06 | 2.23±0.21 | 0.023±0.011 | 0.31±0.02 | 3.51±0.23 |
| *O. arvensis* | | | | | | | |
| 1^st^ week | 0.16±0.01 | 0.016±0.005 | 0.20±0.04 | 1.87±0.28 | 0.010±0.008 | 0.085±0.016 | 2.34±0.23 |
| 2^nd^ week | 0.17±0.02 | 0.010±0.007 | 0.20±0.04 | 2.08±0.38 | 0.013±0.001 | 0.086±0.015 | 2.57±0.45 |
| 3^rd^ week | 0.17±0.02 | 0.010±0.001 | 0.21±0.01 | 1.73±0.09 | 0.012±0.001 | 0.085±0.004 | 2.22±0.10 |
| 4^th^ week | 0.09±0.02 | 0.015±0.009 | 0.13±0.01 | 1.69±0.25 | 0.008±0.001 | 0.059±0.004 | 2.01±0.28 |

## Table S2: The mean absolute isoflavonoid content of *O. spinosa* and *O. arvensis* HRC extracts in mg (n=3)

|  | Pseudobaptigenin glucoside  (*mg*) | Formononetin glucoside  (*mg*) | Sativanone glucoside  (*mg* | Medicarpin glucoside (*mg*) | Sativanone  (*mg*) | Medicarpine  (*mg* | Total  (*mg*) |
| --- | --- | --- | --- | --- | --- | --- | --- |
| *O. spinosa* | | | | | | | |
| 1^st^ week | 0.14±0.051 | 0.031±0.02 | 0.53±0.27 | 1.3±0.56 | 0.02±0.013 | 0.2±0.11 | 2.25±1.03 |
| 2^nd^ week | 0.14±0.015 | 0.030±0.01 | 0.54±0.14 | 1.4±0.22 | 0.020±0.0036 | 0.21±0.056 | 2.32±0.45 |
| 3^rd^ week | 0.21±0.05 | 0.079±0.026 | 1.37±0.59 | 3.0±1.0 | 0.03±0.010 | 0.5±0.23 | 5.20±1.91 |
| 4^th^ week | 0.12±0.03 | 0.051±0.006 | 0.78±0.14 | 2.2±0.44 | 0.02±0.010 | 0.31±0.054 | 3.49±0.62 |
| *O. arvensis* | | | | | | | |
| 1^st^ week | 0.032±0.005 | 0.0029±0.0007 | 0.05±0.015 | 0.5±0.19 | 0.002±0.0009 | 0.021±0.0057 | 0.58±0.20 |
| 2^nd^ week | 0.05±0.03 | 0.0028±0.0018 | 0.059±0.04 | 0.6±0.33 | 0.004±0.0025 | 0.03±0.014 | 0.73±0.41 |
| 3^rd^ week | 0.086±0.02 | 0.005±0.001 | 0.10±0.010 | 0.86±0.08 | 0.0059±0.00034 | 0.042±0.0038 | 1.10±0.11 |
| 4^th^ week | 0.026±0.004 | 0.0042±0.0023 | 0.039±0.0018 | 0.47±0.06 | 0.0023±0.00024 | 0.0166±0.00068 | 0.56±0.06 |
